# The Effect of Film Thickness on the Gas Sensing Properties of Ultra-Thin TiO_2_ Films Deposited by Atomic Layer Deposition

**DOI:** 10.3390/s18030735

**Published:** 2018-03-01

**Authors:** Rachel L. Wilson, Cristian Eugen Simion, Christopher S. Blackman, Claire J. Carmalt, Adelina Stanoiu, Francesco Di Maggio, James A. Covington

**Affiliations:** 1 Christopher Ingold Laboratories, Department of Chemistry, University College London, 20 Gordon Street, London WC1H 0AJ, UK; rachel.wilson.14@ucl.ac.uk (R.L.W.); c.j.carmalt@ucl.ac.uk (C.J.C.); francesco.dimaggio@ucl.ac.uk (F.D.M.); 2 National Institute of Materials Physics, Atomistilor, 405A, P.O. Box MG-7, 077125 Bucharest-Măgurele, Romania; simion@infim.ro (C.E.S.); stanoiu.adelina@yahoo.com (A.S.); 3 School of Engineering, University of Warwick, Coventry CV4 7AL, UK; j.a.covington@warwick.ac.uk

**Keywords:** gas sensing, sensors, ALD, TiO_2_, titanium isopropoxide, thin films, debye length

## Abstract

Analyte sensitivity for gas sensors based on semiconducting metal oxides should be highly dependent on the film thickness, particularly when that thickness is on the order of the Debye length. This thickness dependence has previously been demonstrated for SnO_2_ and inferred for TiO_2_. In this paper, TiO_2_ thin films have been prepared by Atomic Layer Deposition (ALD) using titanium isopropoxide and water as precursors. The deposition process was performed on standard alumina gas sensor platforms and microscope slides (for analysis purposes), at a temperature of 200 °C. The TiO_2_ films were exposed to different concentrations of CO, CH_4_, NO_2_, NH_3_ and SO_2_ to evaluate their gas sensitivities. These experiments showed that the TiO_2_ film thickness played a dominant role within the conduction mechanism and the pattern of response for the electrical resistance towards CH_4_ and NH_3_ exposure indicated typical *n*-type semiconducting behavior. The effect of relative humidity on the gas sensitivity has also been demonstrated.

## 1. Introduction

Semiconducting metal oxides (SMO) have been applied in solid state gas sensors due to their low cost, simplicity of fabrication and use, versatility in detecting a wide range of toxic/flammable gases, and stability in harsh environments. These sensors rely on reversible changes in electrical conductivity upon adsorption/desorption of gas molecules at their surfaces. Interaction with ambient oxygen in an *n*-type SMO can trap mobile carriers (electrons) from the bulk, resulting in an electron-depleted layer (i.e., space-charge layer) at the material surface [1]. If the size of the sensing element is comparable with the depth of the electron-depleted layer, then theoretically the entire sensing element becomes electron-depleted, providing the least conducting state (i.e., greatest change in electrical conductivity) [2]. In the theory of space-charge layers, where the Shockley approximation is still valid, the Debye length represents a measure of surface phenomena screening by the bulk [3]. Sysoev et al. reported the Debye length for TiO_2_ to be ~10 nm at 250 °C [4]. 

Previously, reported methods for depositing thin film gas sensing materials include magnetron sputtering [5], sol-gel [6], chemical vapor deposition (CVD) [7], ultrasonic spray pyrolysis [5] and more recently by atomic layer deposition (ALD) [8]. ALD is a gas-phase thin film deposition technique that involves sequential, alternative dosing of chemical precursors. Precursors chemisorb to the substrate in a non-reversible manner, forming saturated layers. A gas purge step is performed between each precursor dose to ensure film growth occurs via a self-limiting mechanism. Reaction cycles are repeated until the desired film thickness is achieved, where the ultra-thin films deposited are extremely conformal and continuous to the underlying substrate. ALD allows atomic level control of film growth, allowing fabrication of materials with defined thickness and is capable of depositing semiconductor materials in the order of the Debye length, making it an ideal tool for exploring the fundamental sensing properties of these materials. The use of ALD for deposition of gas sensing materials has recently been reviewed [8] but the most persuasive demonstration of the use of ALD for exploring the thickness dependence of sensor response has been the work of Du and George for SnO_2_ [3] where an optimum sensitivity was found at a film thickness of 3 nm, which was suggested to correlate with the Debye length of the material under the test conditions.

**Figure 1:**
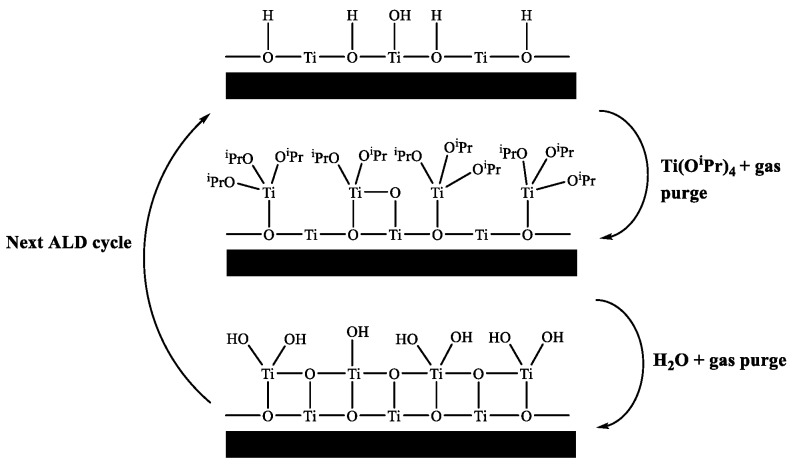
Schematic of ALD growth mechanism using titanium isopropoxide and water.

ALD of semiconducting TiO_2_ is well reported in the literature and has been deposited using a variety of different precursors, such as titanium ethoxide (Ti(OEt)_4_) [9], titanium tetramethoxide (Ti(OMe)_4_) [10], titanium iodide (TiI_4_) [11], titanium chloride (TiCl_4_) [12], tetrakis-dimethyl-amido titanium (TDMAT) [13] and titanium isopropoxide (Ti(O^i^Pr)_4_) [14]. Figure 1 shows a schematic of an ALD deposition cycle for titanium(IV) isopropoxide and water.

Although the gas sensing properties of TiO_2_ have been widely investigated, including TiO_2_ deposited via ALD [15,16,17], there are currently very few reported studies on the effect of TiO_2_ film thickness at the order of the Debye length on the sensing properties. It is also worth noting that most previous studies on ALD of gas sensing materials have used bespoke gas sensor substrates, rather than more traditional ceramic sensor platforms, and while we have previously commented on the importance of gas sensor substrate feature design being appropriate for use of nanoscaled sensing materials [18], the use of non-standard designs makes comparison between published work difficult. In this paper, *n*-type TiO_2_ films were deposited onto alumina gas sensors via ALD at 200 °C using Ti(O^i^Pr)_4_ and water as precursors. By variation of the number of reaction cycles, TiO_2_ films of different thicknesses were deposited. The resistance of these films was then measured at variable temperatures in the presence of CO, CH_4_, NO_2_, NH_3_ and SO_2_ to determine the responsivity of these films as gas sensors. It was observed that the sensor response changes with film thickness and gas concentration.

## 2. Materials and Methods 

### 2.1. TiO_2_ Film Preparation

ALD depositions were carried out using [titanium(IV) isopropoxide], Ti(O^i^Pr)_4_ (obtained from Sigma Aldrich, Gillingham, UK) and deionized water. Pureshiled argon (BOC, 99.998%) was used as the carrier gas. Single-sided alumina sensor platforms, in which the heater track and sensor electrodes are separated by an insulating glassy ceramic, were used as obtained (University of Warwick), and microscope slides (super premium, VWR) were cleaned using isopropanol and air dried before use. TiO_2_ thin films were deposited onto these substrates using a flow-type cold-walled ALD reactor constructed of stainless steel that was built in house. Titanium isopropoxide was held at a temperature of 25 °C and was introduced into the reaction chamber by an argon gas stream (100 sccm) to assist the transportation of vaporized precursor molecules. Water was held at a temperature of 5 °C and was introduced into the reaction chamber by means of its own vapor pressure, without any gas assist. The alumina gas sensors were heated inside the reaction chamber on a heated substrate holder, which was held at a constant temperature of 200 °C throughout the deposition process. Films were deposited by alternately exposing the metal precursor and water into the reaction chamber, with an inert gas purge step in between each dose. Therefore, one complete ALD reaction cycle consisted of a 2.5 s Ti(O^i^Pr)_4_ dose, 1 min purge, 2.0 s water dose and 3 min purge. 100 sccm of inert gas was used to purge the reaction chamber in between each precursor dose step, resulting in a total reactor pressure of ~8 mbar when running. During the depositions, a microscope slide was also placed inside the reactor to provide a sample on which to perform standard film characterization techniques. 

TiO_2_ film thickness was measured using a Semilab SE-2000 ellipsometer and Atomic Force Microscopy (AFM) measurements were performed using a Nanosurf easy Scan Atomic Force Microscope, with a 10 μm tip in non-contact mode with oscillating probe. Scan areas were 5 × 5 μm with measurements taken at 20 nm intervals. All images were set to a physical scale factor of 15 in the z axis. X-ray Diffraction (XRD) measurements were performed using a Bruker-Axs D8 (GaDDS) diffractometer, which operates with a Cu X-ray source, monochromated (Kα_1_ and Kα_2_) and a 2D area X-ray detector with a resolution of 0.01°. X-ray Photoelectron Spectroscopy (XPS) analysis was performed using a Thermo Scientific K-Alpha X-ray Photoelectron Spectrometer with monochromated Al K alpha radiation, a dual beam charge compensation system and constant pass energy of 50 eV. All the peaks reported were charge corrected using C 1s peak position at 284.8 eV as the reference point.

### 2.2. Gas Sensor Testing

**Figure 2:**
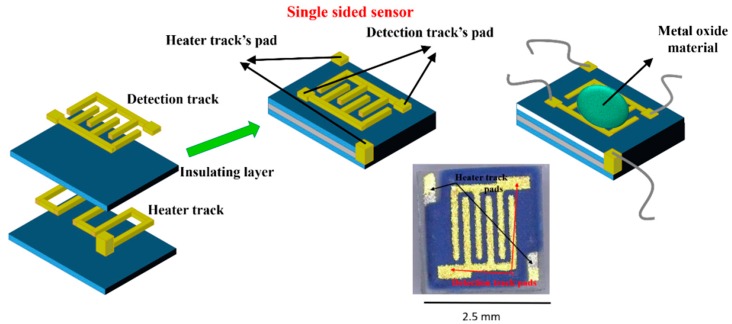
Substrate construction and photo of final device.

**Figure 3:**
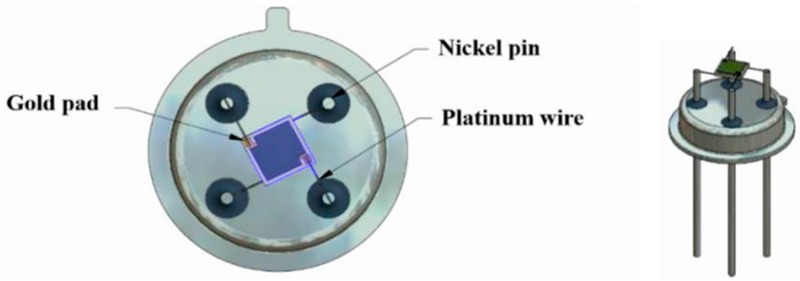
TiO_2_ gas sensor substrate welded onto a TO-39 housing using platinum wires.

Alumina sensors (2.5 × 2.5 mm in size, and 0.550 mm thick, Figure 2) possessed detection electrodes and heater tracks, which were made with gold and platinum materials respectively. The detection electrodes were exposed to air whilst the heater tracks were embedded in a layer of glassy ceramic insulating material, with only the pads exposed for subsequent attachment of wires (Figure 3). A shadow mask was used for deposition, where tungsten foil (100 μm thick) was machined with a 2000 µm diameter hole. After deposition of TiO_2_ the gas sensor substrates were welded onto a TO- 39 housing using platinum wires for interfacing with test equipment.

**Figure 4:**
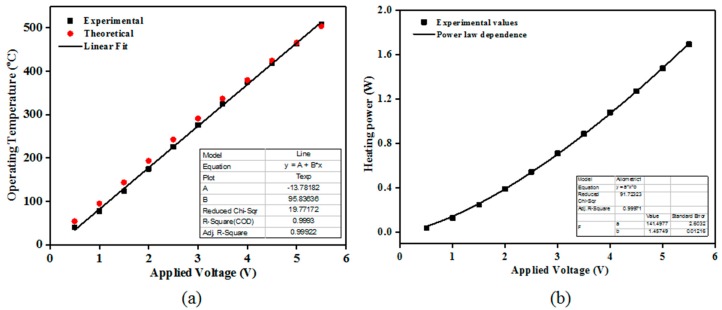
Calibration curves of the operating temperature (**a**) and heating power (**b**) with respect to the applied voltage (experimental and theoretical) for the TiO_2_ coated gas sensor substrates. The emissivity (ε) was set to: 0.76 whereas the theoretical dependence was calculated based on the Pt temperature coefficient (α): 3.92 × 10^−3^/°C.

The electrical resistance of the TiO_2_ films (of different thicknesses’) decreased with increasing temperature (from TΩ to GΩ). This is a general behavior for most of the SMO class materials. It was previously reported that TiO_2_ requires a high operating temperature to accomplish in-field gas sensing demands [19]. Here, the operating temperature was varied from ambient to 480 °C. To keep the operating temperature constant during the gas sensing performance evaluation, the heater calibration of the TiO_2_ coated sensor substrates was performed using a digital pyrometer from LumaSense IN 5-L plus (US) The “hot spot” size was set to 2 mm^2^. Figure 4a shows the experimental and the theoretical dependence of the $T_{heater}=f\left(V_{heater}\right)$ along with the associated linear fitting. 

The associated power consumption at 480 °C was 1.5 W. The power consumption (*P*) obeys the following relation, were *U* represents the applied DC voltage to the Pt heater terminals, *R* is the Pt resistance at temperature *T*; *R*_0_ is the Pt resistance at room temperature; α represents the temperature coefficient of resistance for the Pt heater and Δ*T* is the temperature variation.

(1) $$P=\frac{U^{2}}{R}=\frac{U^{2}}{R=R_{0}\left(1+\alpha \Delta T\right)}$$

As such, one can see that the operating temperature follows a linear dependence with respect to the applied voltage whereas the heating power exhibit a power law behavior (Figure 4b) dependence on the applied voltage with an exponent close to 1.4.

**Figure 5:**
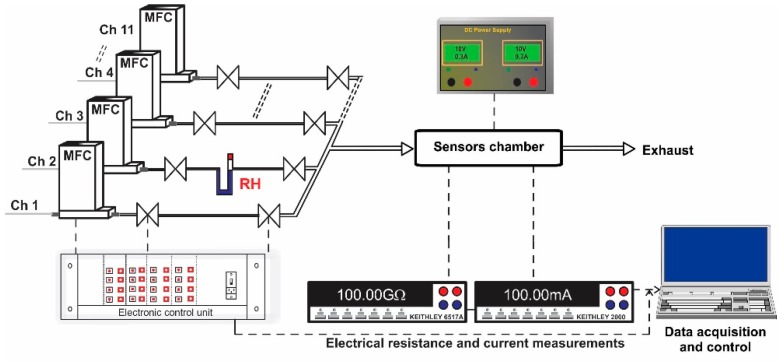
Experimental setup used for gas sensing performance evaluation. Real time acquiring electrical resistance and current data.

The experimental setup consisted of a fully computer-controlled Gas Mixing System (GMS), PTFE sensor chamber designed for TO-39 samples, power supply, Keithley 6517A-Electrometer (US) and Keithley 2000-Multimeter (US) (Figure 5). The GMS has eleven channels provided with high purity (5.0) certified test gases from cylinders. Each gas port was electronically controlled by a Bronkhorst (Netherlands) mass flow controller and two electrovalves. The first two channels were dedicated for carrier gas with controlled relative humidity (RH) in the range of 0–80%. To avoid outgassing, the gas channel interconnections were made of PTFE and stainless-steel with Kalrez seals. The required test gas concentrations were acquired by controlling the flow ratio between the carrier gas and the target gas. The concentrations of the target gases used were: CH_4_ (500, 1000, 1500, 2000 and 2500 ppm) and NH_3_ (50, 70, 100, 150, 200 ppm) dosed in: 0, 10, 30 and 50% RH. The length of each gas pulse was set to 15 min while the dedicated recovery time was set to 30 min. The flow through the system was kept constant at 200 sccm. The sensor signal was calculated according to the relation $S=\frac{R_{air}}{R_{gas}}$ for reducing gases and $S=\frac{R_{gas}}{R_{air}}$ for NO_2_ (oxidizing gas), where *R_air_* is the electrical resistance under dry air and *R_gas_* is the electrical resistance under test gas. For the current passing through the heater, the associated variations have been calculated using the formula: $I=\frac{I_{gas}-I_{air}}{I_{air}}\times 10^{6}$.

## 3. Results and Discussion

### 3.1. Analysis of TiO_2_ Films

**Figure 6:**
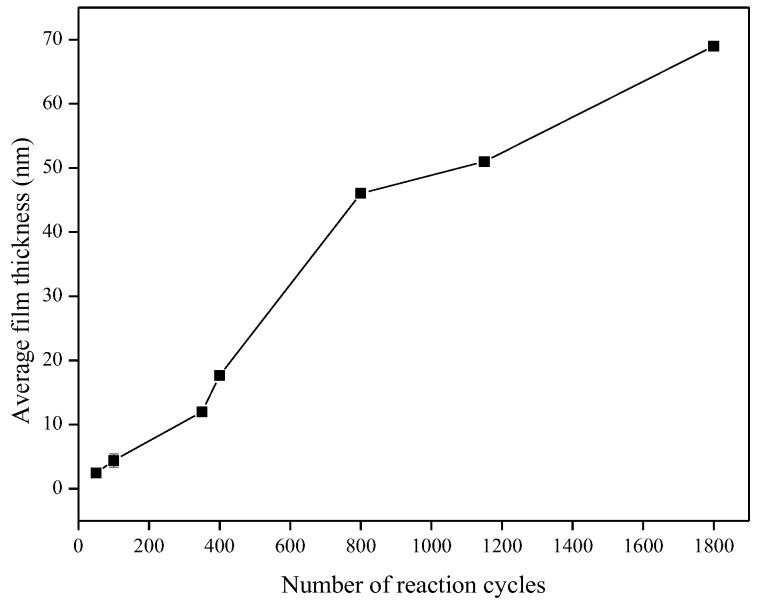
Increase in TiO_2_ film thickness with number of reaction cycles.

**Figure 7:**
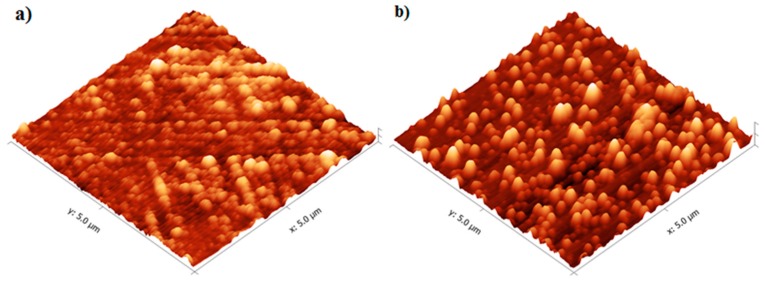
AFM Images of (**a**) 10 nm and (**b**) 50 nm TiO_2_ films deposited at 200 °C.

Characterization of the TiO_2_ films was performed using the films deposited onto microscope slides, placed inside the reactor and deposited simultaneously with the alumina sensors. Figure 6 shows that the TiO_2_ film thickness increased with the number of reaction cycles in an approximately linear fashion, as expected for ALD-like growth where the amount of material deposited in each reaction cycle should be constant. The growth rate was ~0.40 Å/cycle, which is comparable to the growth rate achieved using similar TiO_2_ systems. For the Ti(O^i^Pr)_4_ and water precursor combination, Ritala and Leskelä reported a growth rate of 0.3 Å/cycle in the temperature range of 250–325 °C [20] and Matero et al., reported a growth rate in the range of 0.33–0.60 Å/cycle at 300 °C [21]. The refractive indices of the deposited films (obtained by ellipsometry at 560 nm) were 2.0, 2.3 and 2.8 for films of approximately 10 nm, 50 nm, and 70 nm films respectively. These values were in line with those reported in the literature, which range from 2.3 to 2.6 depending on the deposition temperature [14,22,23]. The refractive index increased with film thickness, which suggests that the density and/or crystallinity of the films increased with thickness. The surface morphology of the TiO_2_ films was studied in more detail using non-contact mode Atomic Force Microscopy (AFM). All films showed good thickness uniformity across the substrate with roughness values similar to the uncoated substrate. Figure 7 shows that as the number of ALD reaction cycles increases, the surface roughness also increases. The root mean square (RMS) roughness values measured for these films are shown in Table 1. 

#### 3.1.1. X-ray Diffraction

**Figure 8:**
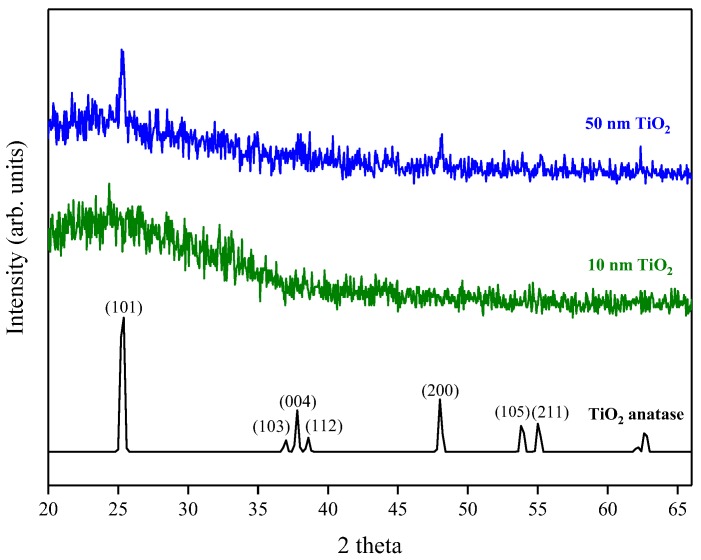
XRD pattern for 10 nm and 50 nm TiO_2_ films deposited by ALD. A typical anatase reference pattern is also included [PDF 01-071-1166].

X-ray Diffraction (XRD) measurements gave no intense peaks for the 10 nm film, which was likely because the film was too thin to produce significant diffraction. This is consistent with the appearance of a broad background peak attributed to break-through to the glass substrate. The 50 nm film, however, showed the presence of peaks attributable to anatase (Figure 8), with the (101) reflection being the most intense (PDF reference number PDF 01-071-1166). As the film thickness increases further the relative peak intensities of the (101) and (200) reflections increased compared to the 50 nm film and the (103), (004), (112), (105) and (211) reflections became more apparent. It has previously been suggested that initially, when the film thickness is low, an amorphous/poorly crystalline layer forms on the surface of the substrate and it is only after subsequent growth that the nucleation sites start to coalesce and form a denser film and become more crystalline [20]. This is consistent with the findings from ellipsometry, where the measured refractive index increased with increasing film thickness. The crystallite size for the 50 nm film was calculated at ~40 nm using the Sherrer equation [24], comparable to the grain size observed using AFM, suggesting that the film consists of a single layer of separated crystallites.

#### 3.1.2. X-ray Photoelectron Spectroscopy

**Figure 9:**
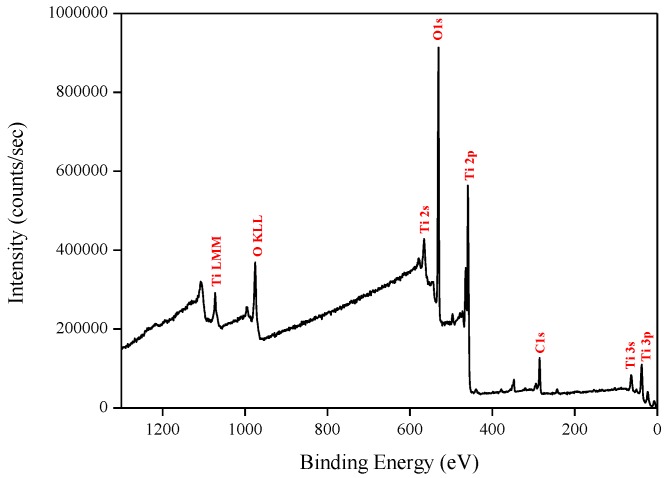
Typical XPS survey of TiO_2_ films deposited by ALD at 200 °C.

**Figure 10:**
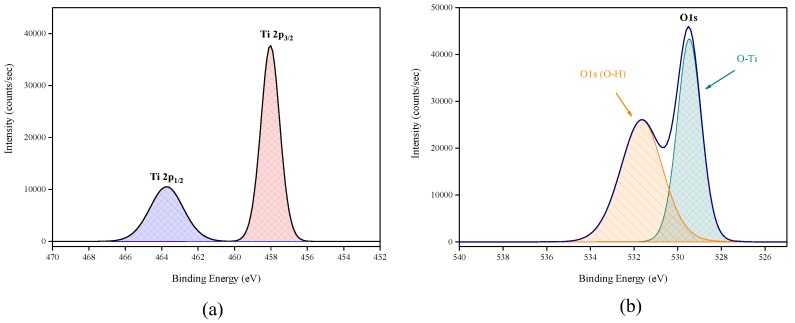
High resolution XPS spectra of (**a**) Ti2p peak and (**b**) de-convoluted O1s peak.

X-ray photoelectron spectroscopy (XPS) data revealed the presence of Ti and O elements on the film surface with minimal contaminants presence, as shown by the survey spectrum (Figure 9). High resolution surface scans (Figure 10a) of the Ti2p peak confirm the presence of Ti^4+^. The 2p_3/2_ and 2p_1/2_ peak binding energies were 458.0 eV and 464.0 eV respectively, with a peak separation of 5.7 eV. These values were within ±0.2 eV of literature values [25,26]. De-convolution of the O1s peak revealed 2 peaks. The peak at the highest binding energy (531.7 eV) can be attributed to surface adsorbed water and the peak with the lowest binding energy (529.5 eV) is ascribed to the O1s core peak of O^2−^ bound to Ti^4+^ (Figure 10b). Again, these peaks are consistent with literature values [27]. The Ti:O elemental ratio, determined from the Ti2p and O1s peak (529.5 eV) areas, was 1:2.4, which confirmed the presence of TiO_2_. XPS also confirmed that the films were pin-hole free as no Si was detected from beam break-through to the substrate. 

### 3.2. Gas Sensing Results

**Figure 11:**
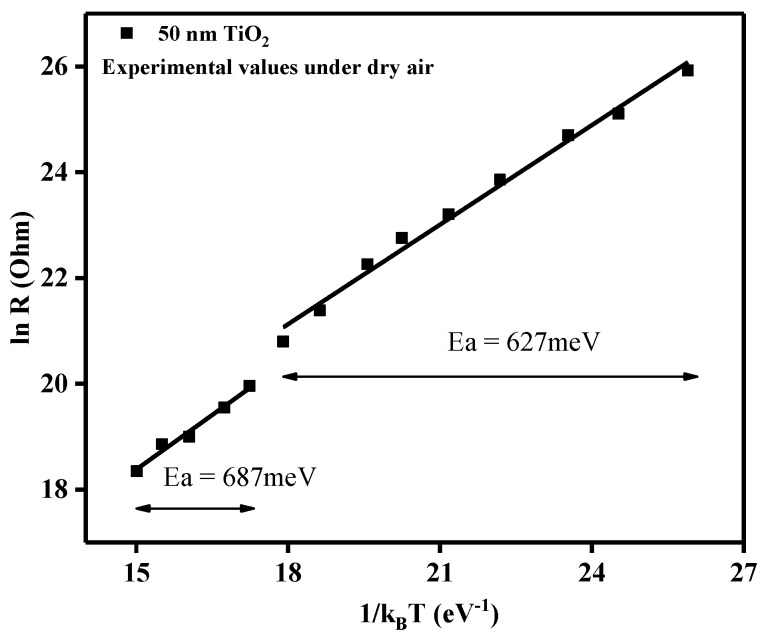
Relation between electrical resistance and operating temperature in the form of ln R and 1/k_B_T.

Prior to sensing property evaluation, the stability of TiO_2_ structures over a temperature range of 175–500 °C was analyzed, measuring the electrical resistance as a function of operating temperature (Figure 11).

Using the plot of the electrical resistance vs. operating temperature for the 50 nm TiO_2_ gas sensor under dry air conditions, we could gain insights about the activation energies according to the Arrhenius equation:

(2) $$lnR=lnR_{0}+Ea\times \frac{1}{k_{B}T}$$

From the slopes of the linear regions, the associated activation energies (*Ea*) could be calculated. As such, the decrease in the electrical resistance, with the increase in the operating temperature, can be ascribed to typical semiconductor behavior. In the temperature range 175–375 °C, the obtained activation energy was *Ea* = 627 meV, whereas for the range 400–500 °C, the activation energy was found to be *Ea* = 687 meV. Even though an increase in temperature induces an increase in the activation energy, for electronic conduction of the TiO_2_ material, the small differences between the two temperature regimes indicated the good stability of the sensitive materials through the whole tested temperature range.

**Figure 12:**
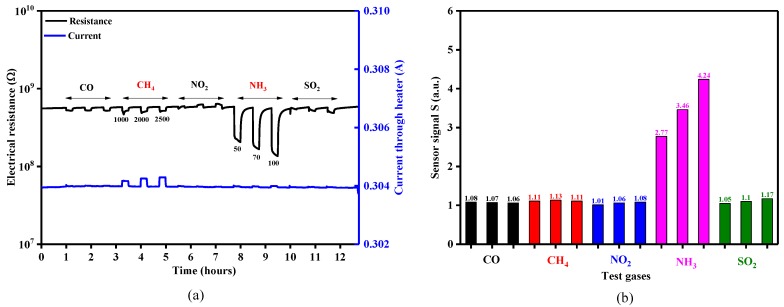
(**a**) Electrical resistance and current response of a 50 nm TiO_2_ coated gas sensor substrates exposed to different concentrations of CO, CH_4_, NO_2_, NH_3_ and SO_2_ under 0% RH at 350 °C and (**b**) the associated sensor signals responses.

The sensing behavior of the TiO_2_-coated gas sensors was evaluated with different test gas concentrations of CO (50, 70, 100 ppm), CH_4_ (1000, 2000, 2500 ppm), NO_2_ (3, 5, 7 ppm), NH_3_ (50, 70, 100 ppm) and SO_2_ (5, 10, 20 ppm) under dry air, spanning the temperature range 23–480 °C. The temperature level of 350 °C was the threshold where appreciable electrical resistance effects could be recorded for the gases of interest (Figure 12a). However, it was also observed that by measuring the electrical resistance of the sensitive TiO_2_ material and the current passing through the platinum heater simultaneously, that CH_4_ exposure also induced a variation in the heating current (Figure 12a—blue line) [28]. As can be seen in Figure 12b, the sensor signals for CO, CH_4_, NO_2_ and SO_2_ exposure were rather negligible compared to those towards NH_3_ exposure (factor up to 4.24). According to the aforementioned reasons, NH_3_ (electrical resistance) and CH_4_ (heater current) were chosen for further investigation. 

**Figure 13:**
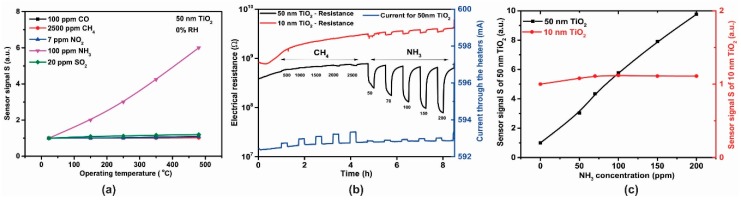
(**a**) Temperature dependence of sensitivity for 50 nm TiO_2_ sensor after exposure to CO, CH_4_, NO_2_, NH_3_, SO_2_, (**b**) Electrical resistance and current response of TiO_2_ gas sensors exposed to different concentrations of CH_4_ and NH_3_ under 0% RH at 480 °C and (**c**) Sensor signal for 10 nm and 50 nm thickness TiO_2_ sensors.

Raising the temperature to 480 °C induced an increase in the electrical resistance effects (Figure 13a) and hence further tests were conducted at this higher temperature. The dynamic responses in electrical resistance changes with CH_4_ and NH_3_ gas exposure (Figure 13b) indicate a typical *n*-type semiconducting-like behavior [29,30]. The influence of TiO_2_ thickness toward the ammonia sensor signals is highlighted in Figure 13c. 

**Figure 14:**
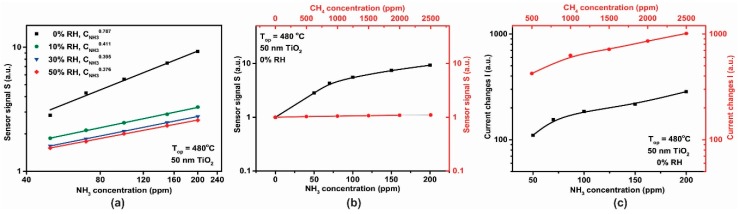
(**a**) Logarithmic plot of sensor signal dependence with respect to the NH_3_ concentration, (**b**) sensor signal dependence and (**c**) heater signal dependence with respect to the NH_3_ and CH_4_ concentrations for the TiO_2_ sensor operated under the same testing conditions 0% RH at 480 °C.

For the 50 nm thick TiO_2_ film, calibration curves for the sensor signal *S* with respect to different NH_3_ concentrations are shown in Figure 14a. The dependence of the sensor signal, $S=R_{air}/R_{NH3}$, dependence on NH_3_ concentration (in the range 50–200 ppm) at 0% RH was fitted to a power law dependence and the associated exponent $C_{NH3}^{x}$ value of 0.787 is similar to that previously reported in the literature [30]. We note that the effect of increasing relative humidity (RH), in the range 0–50%, decreased the NH_3_ sensitivity, which may be related to a competition between NH_3_ and OH^-^ groups for the same pre-adsorbed oxygen species [31]. On the other hand, (Figure 14b) under the same testing conditions, the 50 nm TiO_2_-based gas sensor showed a good selectivity towards ammonia (black curve) vs. methane (red curve).

Electrical current changes in the heater were also observed when the sensors were exposed to CH_4_ (in the range 500–2500 ppm) and NH_3_ (in the range 50–200 ppm) as shown in Figure 14c. Both, the TiO_2_-coated gas sensor and the platinum (Pt) heater have been operated under constant potential mode (U = constant). Based on the metal (Pt) electrical resistance dependence with the temperature, $R=R_{0}\;\left[1+\alpha \left(T-T_{0}\right)\right]$ and with respect to Ohm’s law, the increase in the electrical current passing through the heater during CH_4_ exposure can be translated into a decrease in the electrical resistance (solely at U = constant). A possible explanation of this observation is that we are seeing our sensor behave as a thermal conductivity sensor [32]. As such, we observe a physical detection process, rather than a chemical one. In these sensors, heat is transferred from a hot body to a cold element, thus from the hot gas sensor to the gas it isbeing exposed to. As a gas sensor, thermal conductivity detection can only be used for gases that have a thermal conductivity significantly greater than air. Of these CH_4_ is one of the most important, with a thermal conductivity of 84.1 mW/mK at 350 °C [33]. The change in heater current on exposure to NH_3_ is nearly an order of magnitude smaller than that for CH_4_ (Figure 14c), therefore due to the dual functioning principle of the sensor (electrical resistance and current passing through the heater measurements) the accuracy in the selective-sensitivity is enhanced. Thus, for NH_3_ detection one can focus solely on the resistance variation of the TiO_2_ material whereas for CH_4_ detection the changes within the current passing through the heater are required. 

**Figure 15:**
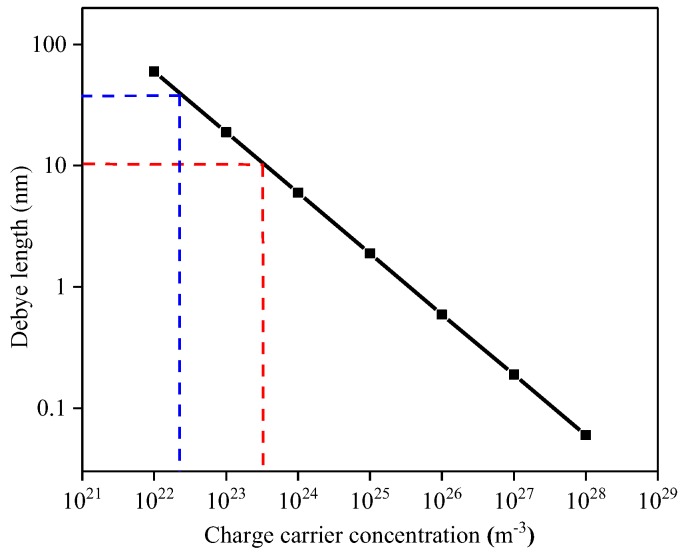
Debye length dependence on the carrier concentration. Red lines are related to the 10 nm TiO_2_ film whereas blue lines are related to the 50 nm TiO_2_ film.

It is evident that the TiO_2_ film thickness plays a dominant role within the conduction mechanism. Reported carrier concentrations for TiO_2_ are in the range from 10^22^ to 10^26^ m^−3^ [34]; Figure 15 correlates the variation of Debye length as a function of charge carrier concentration. The Debye Length dependence was calculated according to relationship:

(3) Ld=ϵε0kBTq2nb

where the operating temperature *T*, of the TiO_2_-coated gas sensor substrates was set to 753.15 K and the dielectric constant ε was set to 18.9 [35]. Since the tested TiO_2_ materials are quite resistive it is reasonable to assume that the charge carrier concentration is at the lower end of those reported in literature, i.e., 10^22^ m^−3^ and hence the Debye length is likely to be within the range of the film thickness examined in this study, i.e., between 10 and 50 nm. We note that our attempts to experimentally measure the carrier concentration were not successful due to the relatively high resistivity of the samples. 

Given that the 10 nm thick film is near the expected value for the Debye length [4] it is perhaps surprising that the sensitivity towards CH_4_ and NH_3_ is so low. It is, however, worth noting that because electrical conduction must take place parallel to the substrate, in the case of the 10 nm thick film, the film can be described as a fully depleted layer due to the ratio between the Debye length and film thickness and hence the lack of sensitivity might be related to the screening effect exhibited by the Debye length.

## 4. Conclusions

We describe the deposition of TiO_2_ films onto alumina gas sensor substrates via ALD of titanium(IV) isopropoxide and water, with thickness values in the range of the expected Debye length. These films were not sensitive to CO, NO_2_ or SO_2_ but were sensitive to NH_3_ and CH_4_, with power exponents typical of the materials. Through simultaneous measurement of the electrical resistance and current through the heater at a constant temperature it was possible to highlight the selective-sensitivity of the TiO_2_ sensors to CH_4_ and NH_3_ test gases. The films were more sensitive towards NH_3_ than CH_4_; where CH_4_ was found to undergo combustion on the TiO_2_ surface. Sensitivity towards NH_3_ varied with thickness, but surprisingly the film of thickness most consistent with a previously determined value for the Debye length of 10 nm, was not the most sensitive. This lack of sensitivity may possibly be due to screening effects induced by a fully depleted layer.

## Figures and Tables

**Table 1:** Film thickness and roughness measurements of TiO_2_ films deposited at 200 °C.

Number of ALD Reaction Cycles	Film Thickness (nm)	RMS (nm)
Blank microscope slide	-	2.3
350	10	3.0
1150	50	3.8
1800	70	4.5

## Abbreviations

The following abbreviations are used in this manuscript:

DOAJ	Directory of open access journals
ALD	Atomic Layer Deposition
SMO	Semiconducting metal oxides
CVD	Chemical Vapor Deposition
TDMAT	tetrakis-dimethyl-amido titanium
AFM	Atomic Force Microscopy
XRD	X-ray Diffraction
XPS	X-ray Photoelectron Spectroscopy
GMS	Gas Mixing System
RMS	Root men square
RH	Relative Humidity
